# Sickness absence and disability pension three years before and seven years after first childbirth: A Swedish population-based cohort study

**DOI:** 10.1177/14034948221125153

**Published:** 2022-10-26

**Authors:** Krisztina D. László, Pia Svedberg, Petra Lindfors, Ulrik Lidwall, Kristina Alexanderson

**Affiliations:** 1 Division of Insurance Medicine, Department of Clinical Neuroscience, Karolinska Institutet, Stockholm, Sweden; 2 Department of Global Public Health, Karolinska Institutet, Stockholm, Sweden; 3 Department of Psychology, Stockholm University, Sweden; 4 Department for Analysis, Swedish Social Insurance Agency, Stockholm, Sweden

**Keywords:** Sick leave, disability pension, childbirth, women, parity, Sweden, longitudinal, cohort study

## Abstract

**Aims:**

There is a widely held belief, in Sweden and internationally, that women with children are more likely to be on sickness absence (SA) than their nulliparous counterparts. However, empirical findings in the field are limited and inconsistent. We aimed to explore initially nulliparous women’s patterns of SA and disability pension (DP) three years before and seven years after 2009, by later parity.

**Methods:**

We conducted a longitudinal cohort study of nulliparous women in Sweden on 31 December 2009 (*N*=426,918). We compared crude and standardized numbers of SA/DP net days in the three years before (Y_–3_ to Y_–1_) and the seven years (Y_+1_ to Y_+7_) after the date of the first birth in 2010 or 2 July 2010 in the following three groups: (1) women with no childbirth during the seven-year follow-up and an additional nine months (i.e. 7.8 years), (2) women with a first childbirth in 2010 and no additional childbirth during the next 7.8 years, and (3) women with their first childbirth in 2010 and minimum one more during the next 7.8 years.

**Results:**

Women remaining nulliparous had consistently more standardized mean SA/DP days than women giving birth. Compared with women with one birth, women with several births had similar mean numbers of standardized SA/DP days during Y_–3_ and Y_–2_, more during Y_+1_ to Y_+3_ and fewer during Y_+4_ to Y_+7_.

**Conclusions:**

**In contrast to the widely held societal belief, we found that in all years women who gave birth had fewer SA/DP days than those remaining nulliparous.**

## Background

There is a widely held belief, in Sweden and internationally, often expressed in mass-media, by employers and politicians, according to which women with children are more likely to be on sickness absence (SA) than their nulliparous counterparts. Indeed, a large number of studies have shown that SA increases during pregnancy, both when compared with pre-pregnancy levels and with women not giving birth [1–7]. However, findings from studies investigating whether women have higher SA levels (i.e. reduced work capacity due to disease or injury) after giving birth than their nulliparous counterparts are more heterogeneous [8]. Some report that women with children have higher SA levels than women without children [9], while others find no association between having children and SA in their overall study populations [8,10,11], but in specific subgroups, for example, among single women [10,12], young women [9,12] or women with high occupational class [13]. In three independent register-based cohorts and in a twin cohort in Sweden, we have found that women who gave birth had lower combined SA and disability pension (DP) days/year than women who remained nulliparous, except for the pregnancy period [1–5]. Moreover, SA/DP levels were low in all groups. Additional knowledge about SA differentials according to motherhood is important as a higher SA/DP in mothers than in women without children may warrant further policies to support families with children. Conversely, if there is no such difference or if it is in the opposite direction, such policies are superfluous. It is important to inform the debate with such knowledge to prevent any health-related discrimination on the labour market of women with children.

Several factors can contribute to SA differences in women with and without children. First, women’s social and psychological resources (e.g. socioeconomic characteristics, family support or emotional resilience) as well as their mental and somatic morbidity may influence both their propensity to engage in childbirth and their labour market activity. Thus, women who give birth, and especially women giving birth multiple times, are likely to be healthier, socially and emotionally more resourceful and to have lower SA than those not giving birth. In addition, according to ‘role enhancement’ or ‘role expansion’ theories, women with children may acquire multiple, complementary networks and roles (associated with being both a parent and an employee), which in turn may have a positive impact on their health, and thus decrease their SA [14]. In contrast, pregnancy, childbirth and the post-partum period can involve different types of morbidity, both in the short and the long term, and increase SA and/or DP. Furthermore, according to multiple strain theories, carrying the ‘double burden’ of both paid and unpaid work may increase risks of ill-health and SA among women [15–18]. Some have argued that, in order to ease their ‘double burden’, some women with children may have a permissive attitude to SA and may be on SA even though their health condition does not require this [19]. These potential explanations are not mutually exclusive and their importance likely varies between women and over time. Women with children are likely to differ from those without children, in their resources, health and risk of SA before their pregnancies. After the first childbirth, and increasingly with additional childbirths, the multiple strains of paid and unpaid work may indeed lead to SA. However, as children grow older, the ‘double burden’ is likely to decrease and support from children may instead become increasingly important.

Comparing levels of SA/DP between nulliparous, primiparous and multiparous women over several years before and after the first birth in parous women is important to understand this complex relationship. While our twin studies followed women for 6–16 years after their first birth [3,4], our previous population-based studies in this area have focused on SA/DP levels in the years close to childbirth, that is, the three years before and the three years after the first delivery date [1,5,20]. The twin studies showed that women not giving birth had higher DP levels both the years before their sisters gave birth and over the following 6-16 years, than those who gave birth even when adjusting by design for familial factors. Moreover, women giving birth only once had higher mean DP levels than women with several births [2,4]. In our previous population-based studies we found, except for the pregnancy period, that the mean SA of women giving birth is generally lower than or comparable to that of women remaining nulliparous [3,4]. Yet, it is unclear whether findings from the long-term follow-up of the Swedish-born twin cohort generalize to women in the general population. We have carried out population-based longitudinal cohort studies of initially nulliparous women aged 16–39 years, selecting study participants with reference to years 1995, 2000 or 2005, and including data from three years before to three years after the birth date [1,5,20,21]. Findings showed that, during the year before pregnancy, women who gave birth had higher levels of SA/DP than women who remained nulliparous. In all other periods, women giving birth had lower SA/DP days than women who remained nulliparous, with SA/DP levels being lowest in women with more than one birth [1,5,20,21]. In order to add knowledge, we aimed to study this for a later time period, also using a longer follow-up than in previous studies. Importantly, and in contrast to several previous studies, DP was included alongside SA.

## Aims

With this study, we aimed to contribute to the societal discussion about SA among women following childbirth. We explored initially nulliparous women’s patterns of SA and DP in the three years before and the seven years after 2009, by later parity.

## Methods

We conducted a population-based longitudinal cohort study by anonymized microdata from five Swedish population-based nationwide registers, linked by the unique personal identification number assigned to all residents in Sweden [22]. The registers used were:

• The Longitudinal Integration Database for Health Insurance and Labour Market Studies (LISA) held by Statistics Sweden [23];
• The Medical Birth Register, the Inpatient Register, and the Cause of Death Register, kept by the Swedish Board of Health and Welfare;
• The Micro Data for Analysis of Social Insurance (MiDAS) database kept by the Social Insurance Agency [24] regarding start and end date and grade (full or part time) of all SA and DP.

Using information from these registers, we identified the study population as all nulliparous women who on 31 December 2009 were aged 18–39 years, registered as living in Sweden during 2007–2009, that is, since at least three years. This resulted in a study population of 426,918 women. We classified these cohort members into three groups based on their childbirths in the period 2010–2017:

• B0: women with no births neither before 2010, nor in the next seven years and nine months (7.8 years);
• B1: women giving birth to their first child in 2010 and no further births in the next 7.8 years;
• B1+: women giving birth to their first child in 2010 and having at least one more birth in the next 7.8 years.

In addition to the seven years, we considered another nine months to exclude any new pregnancy that might have begun during the follow-up years, during which levels of SA would be expected to increase. We obtained information on childbirth and parity from the Medical Birth Register, which includes information on 98–99% of the births in Sweden. We complemented this information on women’s birth by searching for the following birth-related International Classification of Disease (ICD) codes in the Inpatient Register: ICD-7: 660, 670–678; ICD-8: 650–662; ICD-9: 650, 651, 652, 659X,W/659.W-659.X, 669.E,F,G,H,W,X and ICD-10: O75.7–O75.9, O80–84 [1,5,20]. If a birth appeared in both registers, the information from the Medical Birth Register had precedence to that of the Inpatient Register.

For the women who delivered in 2010, we defined the delivery date as the index date (T_0_). For women who remained nulliparous, we considered 2 July 2010 as their T_0_.

### SA and DP insurance in Sweden

All residents in Sweden aged ⩾16 years with income from work or unemployment or parental-leave benefits and who are unable to work due to disease or injury are entitled to SA benefits from the public SA insurance system [25]. Day 1 of a SA spell is a waiting day, with no reimbursement. From day 8, a physician-issued medical certificate is required. For employed individuals, the employer provides sick pay during the first 14 days of a SA spell; from day 15, SA benefits are paid by the Social Insurance Agency. For the unemployed, the Agency pays from day 2 of a SA spell. All residents in Sweden aged 19–64 years can be granted DP if having permanent or long-term work incapacity caused by disease or injury. SA benefits represent approximately 80% of the lost income, DP benefits 65% of the lost income, both up to a certain level. SA and DP benefits may be for full (100%) or part time (25%, 50%, 75%) of ordinary work hours [26]. Thus, individuals can be on part-time SA and DP at the same time.

We obtained information on SA spells >14 days and all DP for the three years before and the seven years after T_0_, that is, for Y_–3_ to Y_+7_ from the MiDAS database. When calculating the duration of the SA, we included also the first 14 days of each SA spell >14 days.

### Other variables

We obtained information on the following demographic factors for the end of 2009 from LISA: age (classified as 18–24, 25–29, 30–34 and 35–39 years); country of birth (Sweden, other Nordic country, other European country, and rest of the world (including missing)); type of living area (large city, medium sized-city, and small town/village, classified according to the H-classification scheme); educational attainment (elementary (⩽9 years), high school (10–12 years) and university/college (⩾13 years)). Study participants with missing information on education were classified as having elementary education.

Years of emigration and death were based on LISA data and the Cause of Death Register, respectively.

### Statistical analyses

We first calculated descriptive statistics for each of the three childbirth groups. Year (Y_–3_ to Y_+7_) was considered in relation to T_0_ (365 days/year, that is, not by calendar year). In all analyses, we combined part-time SA/DP gross days into net days, for example, two days of half-time SA or DP were counted as one net day (hereafter called days). We computed the following measures of SA: number and rates of women with: any SA during a year, with >0–30 days, >30–90 days, >90–180 days, and >180 days/year for each of the 10 studied years for each of the childbirth groups (B0, B1, B1+). Regarding DP, we calculated the numbers and rates of women with DP, with any DP during the year, and on DP all the year, respectively. We also calculated the number and rates of women with any SA or DP during each year. Furthermore, we computed for each of the three childbirth groups the crude and the standardized mean SA and DP net days with 95% confidence intervals (CIs) in each of the three years before and the seven years after their respective index date; T_0_. In the standardized analyses we adjusted for age, country of birth, type of living area and educational level. Women who died or emigrated during the follow-up were censored from the year after the event. Also, we compared the three childbirth groups according to their SA/DP for each specific study year.

We performed analyses using SAS version 9.4.

The project was approved of by the Ethical Review Board in Stockholm (dnr 2007/762-31, 2009/23-32, 2009/1917-32, 2011/806-32 and 2016/1533-32).

## Results

Of our cohort of 426,918 women, 383,511 (90%) remained nulliparous during the study period (B0), 9039 (2%) had their first childbirth in 2010 and had no additional births during the study period (B1), while 34,368 (8%) had their first child in 2010 and at least one more childbirth in the subsequent 7.75 years (B1+) (Table I). Compared with women remaining nulliparous (B0), women giving birth in 2010 were less likely to be aged 18–24 years and more likely to be aged 25–34 years and to have college/university education. The proportions of women with any DP at T_0_ were highest in B0 (5.3%) and lowest in B1+ (0.8).

**Table I.** Characteristics of the study cohort of nulliparous women aged 18–39 years in Sweden in December 2009, by future childbirth status.

Covariates	Childbirth group, *n* (%)		
	B0 *n*=383,511	B1 *n*=9039	B1+ *n*=34,368
Age in 2009, in years			
18 to 24	227,845 (59.4)	1946 (21.5)	9063 (26.4)
25 to 29	65,843 (17.2)	2309 (25.5)	13,932 (40.5)
30 to 34	44,711 (11.7)	2682 (29.7)	9323 (27.1)
35 to 39	45,112 (11.8)	2102 (23.3)	2050 (6.0)
Country of birth			
Sweden	32,8107 (85.6)	7536 (83.4)	30,504 (88.8)
Nordic countries, except Sweden	3926 (1.0)	119 (1.3)	286 (0.8)
EU27, except Denmark, Finland and Sweden	9349 (2.4)	259 (2.9)	557 (1.6)
Rest of the world or missing info	42,129 (11.0)	1125 (12.4)	3021 (8.8)
Type of living area in 2009			
Big city	167,247 (43.6)	4108 (45.4)	15,383 (44.8)
Medium-sized city	136,944 (35.7)	3091 (34.2)	11,966 (34.8)
Rural area	79,320 (20.7)	1840 (20.4)	7019 (20.4)
Educational level in 2009			
Elementary, 0–9 years	88,586 (23.1)	1195 (13.2)	2847 (8.3)
High school, 10–12 years	178,502 (46.5)	3859 (42.7)	12,659 (36.8)
University/college, >12 years	116,423 (30.4)	3985 (44.1)	18,862 (54.9)
Ongoing DP at T_0_			
No	363,200 (94.7)	8803 (97.4)	34,101 (99.2)
Yes	20,311 (5.3)	236 (2.6)	267 (0.8)
DP extent at T_0_ (full or part time)			
0	363,259 (94.7)	8803 (97.4)	34,102 (99.2)
25%	655 (0.2)	19 (0.2)	15 (0.0)
50%	1444 (0.4)	46 (0.5)	51 (0.1)
75%	235 (0.1)	7 (0.1)	2 (0.0)
100%	17,918 (4.7)	164 (1.8)	198 (0.6)

B0 = no childbirth, B1= first childbirth in 2010 and no more childbirths during follow-up, B1 = first childbirth in 2010 and at least one more during follow-up; T_0_ = index date, that is, date of first childbirth in 2010 for parous women and 2 July 2010 for nulliparous women.

DP: disability pension

**Figure 1.**
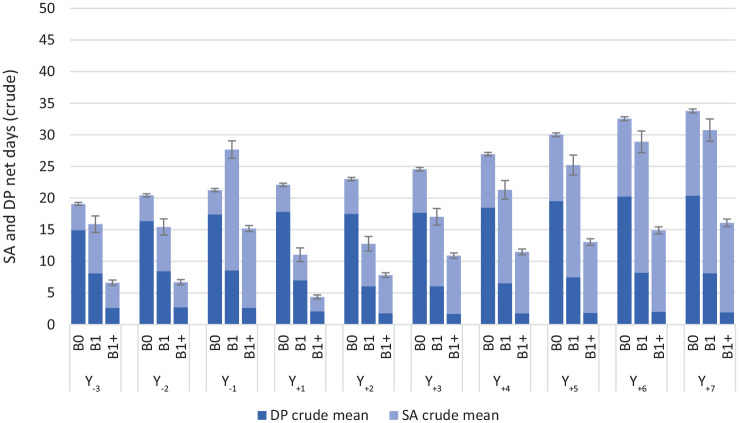
Crude annual mean sickness absence and disability pension net days and 95% confidence intervals for each of the study years in the three childbirth groups. B0 = no childbirth; B1 = first childbirth in 2010 and no more childbirths during follow-up; B1+ = first childbirth in 2010 and at least one more during follow-up; Y_–*n*_ = *n* years prior to the index date (T_0_); Y_+n_ = *n* years after the index date (T_0_); T_0_ = date of first birth in 2010 or, among nulliparous, 2 July 2010. DP: disability pension; SA: sickness absence.

**Figure 2.**
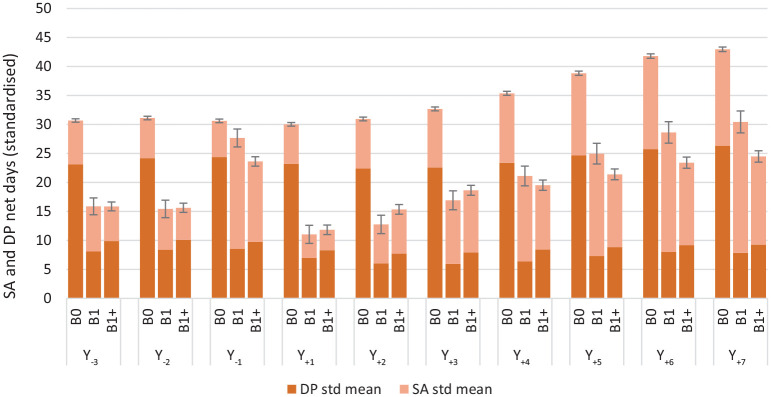
Standardized annual mean sickness absence and disability pension net days and 95% confidence intervals by study year (2007–2016) in the three childbirth groups. B0 = no childbirth, B1 = first childbirth in 2010 and no more childbirths during follow-up, B1+ = first childbirth in 2010 and at least one more during follow-up. Y_–*n*_ = *n* years prior to the index date (T_0_). Y_+n_ = *n* years after the index date (T_0_). T_0_ = date of first birth in 2010 or, among nulliparous, 2 July 2010. DP: disability pension; SA: sickness absence; std: standardized.

Figures 1 and 2 present the crude and the standardized numbers of SA and DP net days per year over the 10 studied years. In general, the SA/DP days per year were few and the CIs narrow. Overall, women in the B0 group had more SA plus DP net days than women in the B1 and B1+ groups, in both the crude and the standardized analyses; this difference was mainly attributable to higher DP rates, and thus more DP days, in the B0 group. In the standardized analyses (Figure 2) there were hardly any differences between the women in the B1 and in the B1+ groups, with three exceptions. In Y_–1_ (that is, when pregnant) the B1 group had significantly more SA days; this was also the case in the three last follow-up years (Y_+5_ to Y_+7_). The third exception is that the B1+ group had significantly more SA/DP days than B1 in Y_+2_. During Y_–3_ to Y_–1_, the B1 group had the highest rates of any SA/DP; after the index date (T_0_), rates of any SA/DP were generally comparable across the three groups (Table II). However, except for the period of the first pregnancy in parous women, SA rates were generally low, overall and for different SA lengths (>0–30, >30–90 days, >90–180 days or >180 days).

**Table II.** Proportion of study participants with different numbers of sickness absence and disability pension net days, by year and childbirth group.

Year	Group	All	On SA and DP		On DP						SA									
			Yes		Yes	Part of year			All the year		Yes		>0–30 days		>30–90 days		>90–180 days		>180 days	
		*n*	*n*	%	*n*	%	*n*	%	*n*	%	*n*	%	*n*	%	*n*	%	*n*	%	*N*	%
Y_–3_	B0	383,511	31,555	8.2	17,722	4.6	3890	1.0	13,832	3.6	15,066	4.1	4786	1.3	4743	1.3	2250	0.6	3287	0.9
	B1	9039	1031	11.4	276	3.1	142	1.6	134	1.5	804	9.0	301	3.4	267	3.0	110	1.2	126	1.4
	B1+	34,368	2112	6.1	343	1.0	185	0.5	158	0.5	1820	5.3	753	2.2	622	1.8	232	0.7	213	0.6
Y_–2_	B0	383,511	33,539	8.7	19,207	5.0	3789	1.0	15,418	4.0	15,269	4.1	5103	1.4	4858	1.3	2213	0.6	3095	0.8
	B1	9039	1064	11.8	283	3.1	138	1.5	145	1.6	820	9.2	341	3.8	284	3.2	93	1.0	102	1.1
	B1+	34,368	2346	6.8	349	1.0	173	0.5	176	0.5	2045	6.0	922	2.7	686	2.0	261	0.8	176	0.5
Y_–1_	B0	383,511	35,193	9.2	20,592	5.4	4410	1.1	16,182	4.2	15,623	4.3	5210	1.4	5139	1.4	2319	0.6	2955	0.8
	B1	9039	2755	30.5	277	3.1	135	1.5	142	1.6	2532	28.5	748	8.4	1187	13.3	454	5.1	143	1.6
	B1+	34,368	7656	22.3	336	1.0	163	0.5	173	0.5	7372	21.6	2608	7.6	3438	10.1	1026	3.0	300	0.9
Y_+1_	B0	383,511	37,894	9.9	21,361	5.6	4878	1.3	16,483	4.3	17,555	4.8	5996	1.6	5819	1.6	2704	0.7	3036	0.8
	B1	9039	866	9.6	241	2.7	119	1.3	122	1.3	649	7.3	354	4.0	172	1.9	71	0.8	52	0.6
	B1+	34,368	1955	5.7	279	0.8	145	0.4	134	0.4	1692	4.9	953	2.8	509	1.5	147	0.4	83	0.2
Y_+2_	B0	377,957	40,572	10.7	20,576	5.4	4545	1.2	16,031	4.2	21,111	5.8	7196	2.0	6621	1.8	3235	0.9	4059	1.1
	B1	9018	912	10.1	202	2.2	95	1.1	107	1.2	728	8.2	303	3.4	216	2.4	105	1.2	104	1.2
	B1+	34,366	3812	11.1	222	0.6	106	0.3	116	0.3	3614	10.6	1431	4.2	1519	4.4	505	1.5	159	0.5
Y_+3_	B0	372,850	44,118	11.8	20,786	5.6	4969	1.3	15,817	4.2	24,748	6.9	7629	2.1	8143	2.3	3893	1.1	5083	1.4
	B1	8947	1175	13.1	204	2.3	105	1.2	99	1.1	1000	11.3	320	3.6	341	3.9	144	1.6	195	2.2
	B1+	34,348	5446	15.9	217	0.6	107	0.3	110	0.3	5264	15.4	2058	6.0	2203	6.4	729	2.1	274	0.8
Y_+4_	B0	368,312	48,623	13.2	21,408	5.8	5101	1.4	16,307	4.4	28,729	8.2	8397	2.4	9156	2.6	4745	1.3	6431	1.8
	B1	8880	1378	15.5	217	2.4	100	1.1	117	1.3	1193	13.6	366	4.2	351	4.0	203	2.3	273	3.1
	B1+	34,324	5021	14.6	223	0.6	103	0.3	120	0.3	4835	14.1	1720	5.0	1985	5.8	754	2.2	376	1.1
Y_+5_	B0	364,155	54,499	15.0	22,423	6.2	5478	1.5	16,945	4.7	33,903	9.8	9288	2.7	10,926	3.1	5744	1.7	7945	2.3
	B1	8822	1567	17.8	246	2.8	114	1.3	132	1.5	1371	15.8	374	4.3	418	4.8	248	2.9	331	3.8
	B1+	34,297	4988	14.5	232	0.7	100	0.3	132	0.4	4787	14.0	1625	4.8	1792	5.2	808	2.4	562	1.6
Y_+6_	B0	360,442	59,071	16.4	22,906	6.4	5535	1.5	17,371	4.8	38,075	11.1	9875	2.9	12,182	3.6	6671	1.9	9347	2.7
	B1	8786	1759	20.0	264	3.0	130	1.5	134	1.5	1549	17.9	404	4.7	503	5.8	251	2.9	391	4.5
	B1+	34,252	5265	15.4	244	0.7	108	0.3	136	0.4	5053	14.8	1602	4.7	1840	5.4	896	2.6	715	2.1
Y_+7_	B0	356,942	62,473	17.5	22,894	6.4	5634	1.6	17,260	4.8	41,644	12.3	11,456	3.4	13,134	3.9	7191	2.1	9863	2.9
	B1	8755	1853	21.2	259	3.0	124	1.4	135	1.5	1649	19.1	423	4.9	502	5.8	312	3.6	412	4.8
	B1+	34,194	5447	15.9	238	0.7	103	0.3	135	0.4	5245	15.4	1624	4.8	1846	5.4	946	2.8	829	2.4

B0 = no childbirth, B1 = first childbirth in 2010 and no more childbirths during follow-up, B1+ = first childbirth in 2010 and at least one more during follow-up. Y_–n_= *n* years prior to the index date (T_0_); Y_+n_= *n* years after the index date (T_0_); T_0_ = date of first birth in 2010 or, among nulliparous, 2 July 2010.

DP: disability pension; SA: sickness absence.

## Discussion

This 10-year population-based longitudinal exploratory cohort study of initially nulliparous women in Sweden showed that in both the crude and the standardized analyses, women who gave birth had fewer combined SA/DP net days per year than women who remained nulliparous; this difference was mainly due to lower DP proportions in childbearing women. Compared with women with one birth during the study period, women with several births had similar SA/DP in their pre-pregnancy years, but higher SA/DP in the first three years after their first birth, while SA/DP levels were lower in the year before and four to seven years after their first birth. Nevertheless, the numbers of SA and DP days were low, as were the rates of women on SA and DP, except for the year of pregnancy.

### Comparison with previous studies

Our findings that SA increased during the first pregnancy as compared with both women’s pre- and post-pregnancy levels are in line with several studies documenting higher SA levels during pregnancy [1–7]. Differences in SA/DP during Y_–1_ between parous women and women not giving birth were somewhat less pronounced in this study than in our previous cohort studies based on data from the nulliparous women in 1995, 2000 or 2005 [1,5,20]. One reason for this may relate to differences in study designs, that is, our longer study period (seven years after T_0_ as opposed to three years in our previous studies) means that more women of those originally nulliparous at study entry gave birth. There are some differences between the B0, B1 and B1+ groups between the studies, for example, the proportion of those in the B0 group is lower and in the B1+ is higher in this study than in the previous studies, in which we followed women for only three years after T_0_. Longer follow-up periods, as well as including information on morbidity may further the understanding of these phenomena, especially regarding the younger women.

Previous findings regarding differences in SA/DP between women with and without childbirths/children have been heterogeneous [8]. The present findings, showing that women with childbirths in general had lower levels of SA/DP than women who remained nulliparous, are in line with previous longitudinal studies with population-based cohorts in Sweden for the years 1995, 2000 and 2005 (focusing only on the three years before and the three years after birth) [1,5,20] and with comparable findings in twin sisters [2–4]. In contrast, other studies (generally combining questionnaire-based data with employers’ or national administrative SA databases and often focusing exclusively on SA) have found that women with children have higher mean SA levels than women without children [9], or that the association between having dependent children and SA was moderated by a third factor [9,10,12,13], while others reported no associations [8,10,11]. In a randomly selected sample of working-aged (i.e. 16–65 years) employees in Sweden in the 1980s, women with young children (<7 years) had a lower risk of having had self-reported repeated short SA spells (defined as three or more SA spells of less than seven days during the year of the interview), but higher risks of long SA spells (defined as at least one spell of more than 59 days per year) than women with no children [27]. In another study of working-aged individuals in Sweden in the 1980s, women up to the age of 34 years with children had a higher SA risk than women without children. However, in older ages this difference disappeared or was even reversed [12]. Another study following SA during 1993–2003 in a cohort of women born in 1969 has in part shown similar findings, that is, women with children had higher SA risk than those without up to the age of 35 years, but there were no SA differences beyond this age; analyses restricted to single women showed that mothers had higher SA than non-mothers irrespectively of their age [9]. Investigating randomly selected municipality female employees aged <50 years in 2000 (*N*=1464), Voss et al. found no SA differences (i.e. ⩽4 SA spells or SA spells ⩾28 days) between women with and without children during the one-year follow-up, except for single mothers who tended to have a higher SA risk than single women without children [10]. A Finnish study found that women with young children had higher SA risk, but only among white-collar employees [13].

Thus, our study, as well as some other studies, indicates the importance of including DP in these types of studies – especially as the time on long-term SA before granted DP can vary immensely between countries, over time and between individuals. In Sweden, SA spells can continue for years, or even decades, before being granted DP, depending on time and practices among social insurance officers.

Our findings regarding the differences in SA/DP among women with one child as compared with those with more than one child are only partly comparable to the findings of our previous population-based studies. In these earlier investigations we found that women with more than one child during the study period had, except in years 2 and 3 after first birth, the lowest levels of SA/DP [1,5,20].

There are several potential explanations for our study findings. A reason for the lower SA/DP, and in particular DP, in the parous groups throughout the whole study period is that women with good health and good social and psychological resources may be more likely to engage in a pregnancy than women with work-limiting health conditions or fewer social and psychological resources. Nevertheless, several women were on DP, even full-time DP, when giving birth in 2010. Additional factors that may contribute to parous women’s lower mean SA/DP days after childbirth versus the nulliparous’ may include role enhancement, that is, having both paid work and being a mother may provide many women with feelings of fulfilment, satisfaction and well-being, which may promote their health [14,28]. As previously reported [26], the fact that women in B1+ had higher or comparable levels of SA/DP in the second and third years after childbirth than those in the B1 group may relate to a next pregnancy. Also, our findings are similar to those of Mastekaasa [14], Åkerlind et al. [12] and Floderus et al. [9], who showed that women with young children had higher SA risks compared with those without children, but that this difference decreased when the children became older. This may potentially reflect the ‘double burden’ [16,17] arising from the combination of paid work and unpaid, domestic work as a possible explanation in women with young children. After the first three years following the first childbirth, women with multiple births had the lowest level of SA/DP of all three childbirth groups. This may support the health selection or role enhancement theories [8] rather than the ‘double burden’ theory [16,17] as potential explanations during this period. However, SA and DP are not optimal ways to measure health or morbidity in cohort studies. Most people with different types of morbidity are not on SA or DP, as the limitations of these morbidities are not severe enough to legitimate SA or DP [29]. A further explanation is that women with several births may be more likely to work part time for a longer time period than those with only one child, which in turn may also lead to fewer days with SA in the former group.

### Strengths and limitations

The strengths of this study include the high coverage of births in Sweden by the Medical Birth Register (98–99%) and the fact that we complemented this information with data on births from the Inpatient Register, which reduced biases related to misclassification in the case of childbirth groups. Moreover, all in Sweden fulfilling the inclusion criteria were included, not a sample. Additional strengths include the high-quality register data leading to no drop-outs and no recall bias while allowing reliable long-term follow-up population data [1,5,20]. Other strengths are the large cohorts, that all SA and DP was medically certified by a physician and that we used several different measures of SA and DP [30]. However, one possible limitation relates to our having information only on SA spells >14 days. This means that the number of SA days are somewhat underestimated; however, this holds for all three study groups. Second, though almost all women who give birth in hospitals in Sweden are recorded in the Medical Birth Register and include information on parity, some women giving birth abroad might have been erroneously classified as nulliparous. However, we expect this misclassification to be small since women not residing in Sweden during the three years preceding 2010 were excluded from the study cohort. Additionally, our findings might not generalize to countries with other SA insurance systems, other quality and coverage of health care, subsidized childcare and women in gainful employment.

## Conclusion

In this large, exploratory cohort study of initially nulliparous women, we found that women who gave birth had lower SA/DP rates and mean days than their counterparts who remained nulliparous; this difference was observed in the two and three years before birth and in the seven-year period after childbirth. Previous findings that women with more than one childbirth have fewer SA/DP days than those with only one childbirth were not replicated here regarding the first years after the first childbirth. In the first three years after the first birth, women with several births had more, and then fewer, SA/DP days per year than women with one birth. The study shows the importance of including both SA and DP when exploring future SA among women giving birth.
